# Study on metastasis inhibition of Kejinyan decoction on lung cancer by affecting tumor microenvironment

**DOI:** 10.1186/s12935-020-01540-0

**Published:** 2020-09-14

**Authors:** Meijuan Chen, Cheng Hu, Qian Gao, Liqiu Li, Ziyu Cheng, Qirui Li, Zhihui Li, Zhaohui Wang, Zejia Mao, Weiqian Tian, Xu Zhang

**Affiliations:** 1 School of Medicine & Holistic Integrative Medicine, Nanjing University of Chinese Medicine, Nanjing, 210023 China; 2 Affiliated Hospital of Nanjing University of Chinese Medicine, Jiangsu Province Hospital of Chinese Medicine, Nanjing, 210029 China

**Keywords:** Lung cancer, Kejinyan decoction, Tumor microenvironment, Tumor-associated microphage (TAMs), Glycolysis, Metastasis

## Abstract

**Background:**

Kejinyan decoction, as an experienced formula of Zhou Zhongying (the Master of Traditional Chinese Medicine) has been widely used in clinic for lung cancer treatment in China, while the anti-lung cancer mechanism of it is still remained to be elucidated. Herein, our basic study found that the survival of lung cancer xenograft mice was significantly prolonged after intragastrically administered high dose of Kejinyan decoction (3.8 g per kg BW) for 15 days. More importantly, we found that Kejinyan decoction inhibited the metastasis of lung cancer cells in vivo. Thus in this study, we aim to elucidate the anti-metastasis effects of Kejinyan decoction.

**Methods:**

RNA-Seq was used to find out the gene regulation of Kejinyan decoction on the mice, flow cytometry assay was used to detect the immunocytes in the spleen, ELISA assay was used to detect the inflammatory factors in the serum and spleen, and immunofluorescence assay was used to detect the level of immune cells and the expression of glycol-metabolism related enzymes in situ. Also, we established a lung cancer orthotopic xenograft tumor model to assess the influence of Kejinyan decoction on the metastatic ability of lung cancer cells in vivo.

**Results:**

GO analysis of gene sequencing of tumor tissue samples showed that Kejinyan decoction regulated immune response. Further flow cytometry analysis of splenic lymphocyte showed that Kejinyan decoction upregulated M1 macrophages and downregulated M2 macrophages, while the total level of macrophages changed little, which was verified by detection of CD68, F4/80, CD206, and CD86 in tumor tissue section. Moreover, detection of inflammatory cytokines showed that Kejinyan decoction downregulated TNF-α, IFN-γ, IL-6, as well as IL-4, IL-13 in tumor microenvironment. Further studies also showed that Kejinyan decoction had little effect on tumor hypoxia, but downregulated glycolysis in tumor tissues. More importantly, we found that Kejinyan decoction inhibited the metastasis of lung cancer cells in vivo.

**Conclusion:**

Our findings conclude that Kejinyan decoction inhibited lung cancer cell metastasis through affecting macrophage polarization and energy reprogramming.

## Background

Lung cancer is the leading cause of cancer death around the world [1]. In China, the incidence of lung cancer increased rapidly, metastasis and recurrence are the main causes of the death of lung cancer. In clinic, poor functioning of immune system usually results in tumor progression and deterioration of cancer patients, while radiotherapy and chemotherapy may lead to further deterioration of the immune function of patients [2, 3].

In recent years, tumor microenvironment (TME) has attracted more and more attention on the treatment of tumor. TME mainly comprises cancer cells and stromal cells, including macrophages, endothelial cells, fibroblasts, and their product [4–6]. Moreover, tumor-associated macrophages (TAMs) are most abundant components in tumor immune microenvironment, which mainly include two polarization subsets M1 (classically activated, pro-inflammatory) and M2 (alternatively activated, immunosuppressive) [7, 8]. Especially, M2 subset is most predominant within tumor, which results in immunosuppressive tumor environment, and promotes tumor cell growth, angiogenesis and immune escape [9, 10]. Furthermore, some cytokines and chemokines produced by tumor cells also promote M2 macrophages polarization, and result in immunosuppressive microenvironment formation in return [9]. So, researchers increasingly realize that improving immunosuppressive TME is beneficial for cancer treatment [11].

Traditional Chinese Medicine (TCM) has been used in clinic for thousands of years in China, especially it plays important roles in prolonging life and improving the quality of life of cancer patients. Professor Zhongying Zhou is the National TCM master in China who creates “cancerous toxin” theory and prefers combining traditional Chinese and Western medicine in cancer treatment. For the treatment of lung cancer, he attached great importance to the combination of strengthening the body and eliminating pathogenic factors. Kejinyan decoction is a basic anti-lung cancer prescription created by him which has good clinical effect on patients. Our previous study verified that this formula inhibited tumor progression in A549 cell xenograft nude mouse model [12], while the anti-lung cancer mechanism remains to be found.

In the present study, RNA-sequencing of the tumors separated from Lewis lung cancer xenograft C57BL/6 mice showed that Kejinyan decoction significantly regulated immune system process and metabolic pathways. Further detection of immune cell subsets by flow cytometry demonstrated that Kejinyan decoction significantly changed the polarization of macrophages. In addition, immunofluorescence detection of macrophage cell surface antigens verified that Kejinyan decoction decreased the level of M2 macrophages, while increased the level of M1 macrophages in the tumor microenvironment. Moreover, detection of the protein level of GLUT1 and FBP1 indicated that the medicine reduced glucose metabolism level in tumor microenvironment. More importantly, the level of inflammatory factors such as IL-4, IL-6, TNF-α, IFN-γ in mice showed a significant downward trend under treatment of Kejinyan decoction. At last, in Lewis lung cancer orthotopic xenograft tumor model, we found that dissemination and metastasis in the luciferase tracking cancer cells were both suppressed by Kejinyan decoction. Collectively, this study may advance our understanding of the anti-lung cancer effect of Kejinyan decoction.

## Material and methods

### Drugs and reagents

Kejinyan decoction is made up of 13 herbs, including *Semen Benincasae*, *Arisaema Cum Bile*, *Houttuynia Cordata*, *Rhizoma Fagopyri Dibotryis*, *Cremastra Appendiculata*, *Herba et Gemma Agrimoniae*, *Carthami Flos*, *Lignum Sappan*, *Rhizoma Pinelliae*, *Radix Ranunculi Ternati*, *Radix Adenophorae*, *Radix Ophiopogonis*, and *Rhizoma Polygonati Odorati*, according to the conventional method (Table 1).

**Table 1:** Main composition of Kejinyan decoction

Main composition	Latin scientific name	Parts used	Amount (g)
Semen Benincasae (Dong gua zi)	*Semen benincasae*	Seed	15
Arisaema Cum Bile (Dan nan xing)	*Arisaema cum bile*	Radix	12
Houttuynia Cordata (Yu xing cao)	*Houttuyniae herba*	Tuber	15
Rhizoma Fagopyri Dibotryis (Jin qiao mai)	*Fagopyri dibotryis rhizoma*	Tuber	15
Cremastra Appendiculata (Shan ci gu)	*Cremastrae pseudobulbus pleiones pseudobulbus*	Radix	10
Herba et Gemma Agrimoniae (Xian he cao)	*Agrimoniae herba*	Herb	15
Carthami Flos (Hong hua)	*Carthami flos*	Flower	10
Lignum Sappan (Su mu)	*Sappan lignum*	Radix	10
Rhizoma Pinelliae (Ban xia)	*Pinelliae rhizoma*	Rhizoma	10
Radix Ranunculi Ternati (Mao zhua cao)	*Ranunculi ternati radix*	Radix	15
Radix Adenophorae (Nan sha shen)	*Adenophorae radix*	Radix	12
Radix Ophiopogonis (Mai dong)	*Ophiopogonis radix*	Radix	12
Rhizoma Polygonati Odorati (Yu zhu)	*Polygonati odorati rhizoma*	Rhizoma	12

The quality control analysis of lyophilized powder of Kejinyan decoction was performed by HPLC [13].

### Cell line and culture conditions

Lewis lung cancer (LLC) cells obtained from the Cell Line Bank (Shanghai, China) were used for the experiments. The cell line was cultured in RPMI-1640 (Gibco, Carlsbad, CA) with 10% fetal bovine serum (Gibco, Carlsbad, CA), 100 U/ml penicillin, and 100 mg/ml streptomycin (Amresco) in a 5% CO_2_ cell culture incubator (Forma, Thermo Scientific, Waltham, MA) at 37 ℃.

### Xenograft experiments

The 5-week-old C57BL/6 mice were maintained under specific pathogen-free (SPF) conditions. Animal welfare and experimental procedures were performed in compliance with the National Institutes of Health Guidelines for the care and use of laboratory animals, and all protocols were approved by the Ethics Review Committee of Nanjing University of Chinese Medicine. LLC cells (2 × 10^7^) were injected subcutaneously. 7 days later, the tumor appeared, after its volume reached approximately 50 mm^3^, the mice were randomly divided into five groups (6 in each group), including the Kejinyan decoction groups (0.95, 1.9 or 3.8 g/kg p.o. daily), and the Normal or Mock group (saline, p.o. daily). After 15 days of treatment, the mice were sacrificed by cervical dislocation, and the tissues were isolated for subsequent experiments.

### ELISA determination of various cytokines in peripheral blood

Mice serum preparation: orbital blood was collected and centrifuged at a speed of 3000 r min ^−1^ for 15 min. The upper serum was taken out of a centrifuge tube and stored at – 70 ℃. Contents of IFN-γ (Lot No.228070832, Multi Science), TGF-β (Lot No.228170744, Multi Science), IL-4 (Lot No.220471131, Multi Science), IL-13 (Lot No.221371135, Multi Science), IL-6 (Lot No.220671132, Multi Science), IL-10 (Lot No.221070945, Multi Science), TNF- α (Lot No.218280443, Multi Science) in serum of mice were detected by sandwich ELISA assay according to the Kit instructions.

### RNA sequencing

Total RNA was extracted using the Illumina Gene Expression Sample Prep Kit (Illumina, Inc., San Diego, CA, USA) according to the manufacturer's protocol. Quality and quantity analyses of total RNA, DGE library preparation, and sequencing were carried out at Allwegene Genomics Co., Ltd. (Beijing, China; Project ID: AWGT18062109). We used a false discovery rate (FDR) ≤ 0.05 and the absolute value of log2 ratio ≥ 1 as the thresholds to judge the significance of gene expression differences [14].

### Immune cell detection of by Flow cytometry

Preparation of the sample: spleens of the mice were grinded and passed through a nylon tissue strainer to obtain a single-cell suspension. 2 ml of the suspension was added slowly into 3 ml ficoll-faque media, spleen leucocytes were obtained by centrifuge at 400*g* for 30 min at 18 ℃. Then the layer of mononuclear cells were transferred to a sterile centrifuge tube using a sterile pipette.

Added at least 3 volume (~ 6 ml) of balanced salt solution to the mononuclear cells in the centrifuge tube, and suspended the cells by gently drawing them in and out of a pipette. Centrifuged at 500*g* for 15 min at 18 ℃, then removed the supernatant. Repeated the above steps again, and resuspended the cell pellet in media appropriate for the application.

Flow cytometry (FCM) was used to analyze the leukocytes: First, the cells were incubated for 30 min at 22 ℃ with the following antibodies: FITC-CD86 (Lot No.561962, BD Pharmingen™), AF647-CD206 (Lot No.565250, BD Pharmingen™), PE-F4/80 (Lot No.565410, BD Pharmingen™), BV421-CD49b (Lot No.563063, BD Pharmingen™), PerCP-CyTM5.5-CD3e (Lot No.561108, BD Pharmingen™), PE-CyTM7-CD11b (Lot No.561098, BD Pharmingen™), then the cells were analyzed by FCM (Beckman), at least 20,000 events were scored and analyzed with Kaluza Analysis v1.3. Single cells were gated for CD4 (Vioblue) and CD3e (APC) to select double-positive cells.

### Immunofluorescence

Frozen sections of lung tumor tissues were fixed with 4% PFA for 15 min, and permeabilized with 0.3% Triton X-100 for 15 min, followed by blockade with 5% goat serum (Beyotime Biotechnology, C0265) for 1 h at room temperature. Then, sections were incubated with primary antibodies overnight at 4 ℃. Secondary antibodies were added, followed by staining with DAPI. Stained sections were imaged using a Laser confocal microscope (TCS SP8, Leica). The primary antibodies used include: anti-mannose receptor antibody ab64693; anti-CD86 antibody ab119857; anti-F4/80 antibody ab6640; anti-Glucose Transporter GLUT1 antibody ab652; anti-FBP1 antibody ab109732. The secondary antibodies used were: Alexa Fluor 647-conjugated AffiniPure donkey anti-rabit IgG (Lot No.142730) and Alexa Fluor 594-conjugated AffiniPure donkey anti-rabit IgG (Lot No.138702).

### Lung metastasis in vivo

To assess the influence of Kejinyan decoction on the metastatic ability of Lewis lung cancer cells in vivo, we established a Lewis lung cancer orthotopic xenograft tumor model. Firstly, we established the luciferase-expressing Lewis lung cancer cell line with lentivirus (Ubi-MCS-firefly_Luciferase-IRES-Puromycin, GENE, Shanghai, China), then Lewis-luc cells (5 × 10^6^ in 0.2 ml medium of a 1:1 mixture of RPMI 1640 and Matrigel 354248) were injected into left lung parenchyma of C57BL/6 mice. Two weeks later, the Lewis lung cancer model mice were identified and randomly divided into three groups that were treated with Kejinyan decoction (1.9, 3.8 g/kg orally (p.o.), 0.2 ml daily; n = 10) and a control group that was treated with saline (control, 0.2 ml, p.o. daily; n = 10) for 30 consecutive days. During this period, we monitored metastasis by luciferase imaging of live animals using an IVIS Spectrum bioluminescence imaging system (PerkinElmer, US) and the intraperitoneal injection of 200 μl D-Luciferin substrate (15 mg/ml in DPBS, PerkinElmer). The protocols for the animal experiments were approved by the Ethics Review Committee of Nanjing University of Chinese Medicine.

### Statistical analysis

Data entry and all analyses were performed in a blinded fashion. All statistical analyses were performed using GraphPad Prism7.0 software. Statistical significance was calculated using two-tailed unpaired *t*-test on two experimental conditions or two-way ANOVA when repeated measures were compared, with *p* < 0.05 considered statistically significant. All graphs show mean values ± SEM.

## Results

### Kejinyan decoction prolonged the survival of Lewis lung cancer mice

**Fig. 1:**
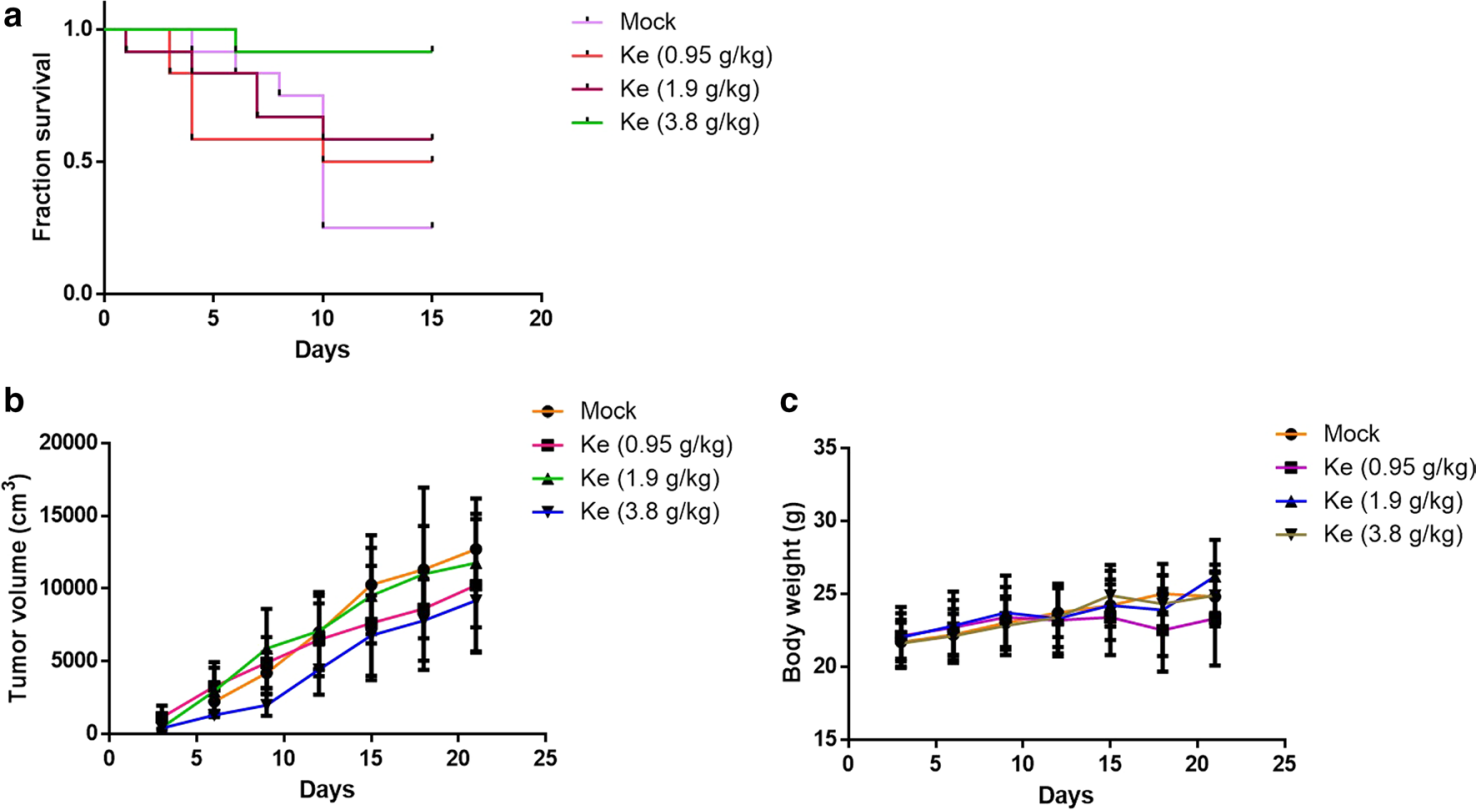
Effects of Kejinyan decoction on the survival of Lewis lung cancer cell xenograft C57BL/6 mice. **a** Survival time of the mice treated with different concentrations of Kejinyan decoction. **b** Tumor volume of the mice in different groups. **c** Body weight of the mice in different groups. Values are means of three separate experiments ± SD

In the Lewis lung carcinoma C57BL/6 mice, we found that Kejinyan decoction significantly improved the quality of life of the mice, and prolonged the overall survival of the mice especially at the high dose of Kejinyan decoction (3.8 g/kg) (Fig. 1a). While the effect on tumor proliferation was not obvious (Fig. 1b, c).

### Overview of the differentially expressed genes between Kejinyan decoction treated group and the mock group

**Fig. 2:**
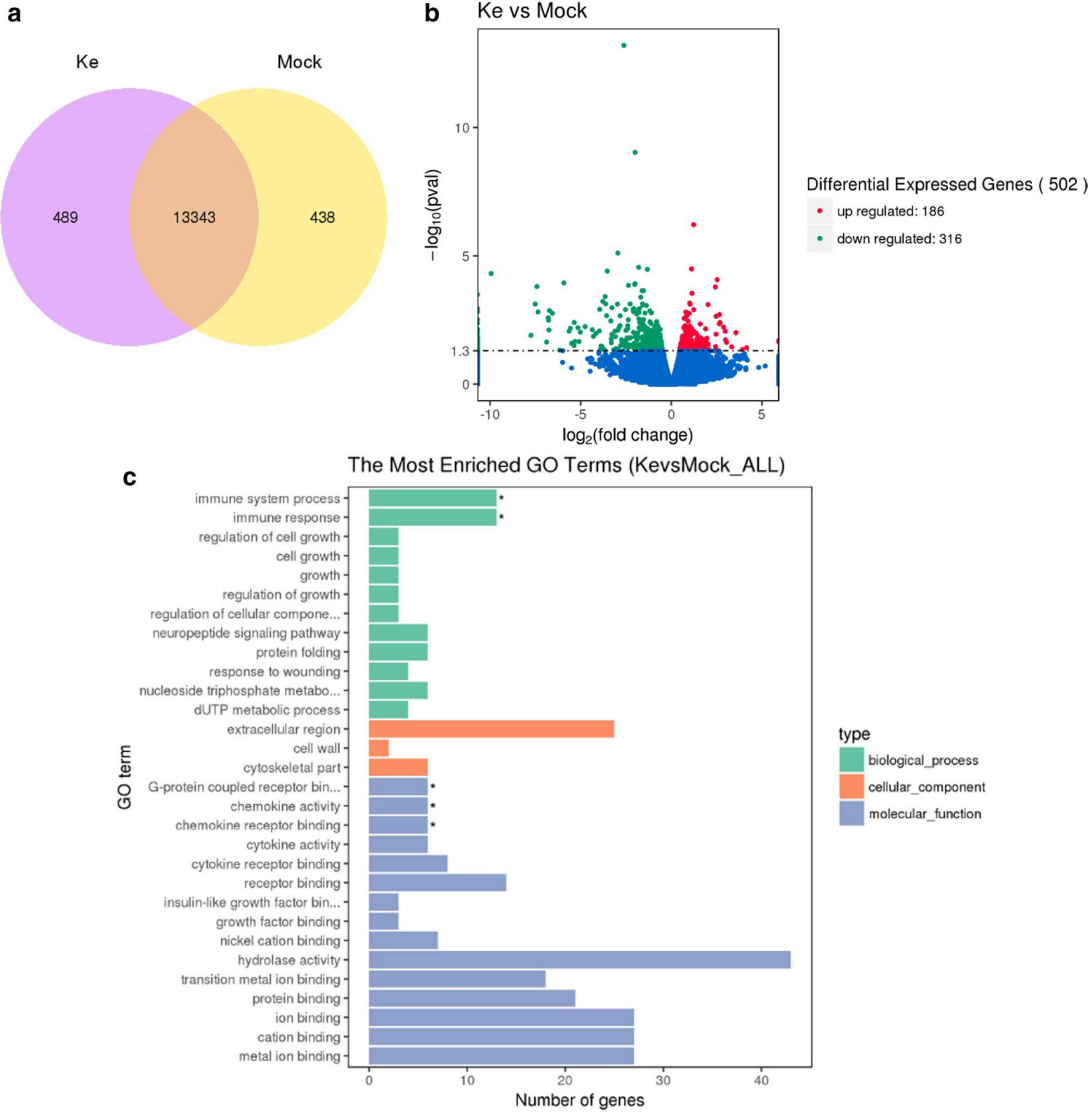
Differentially expressed transcripts (DETs) between the Mock group and the high dose of Kejinyan decoction group. **a** Common genes were identified between the two groups through a Venn diagram. **b** DETs between the Mock group and the high dose Kejinyan decoction group (|log2(FoldChange)|> 1, q value < 0.05). **c** Functional analysis of differentially expressed genes based on RNA-Seq Data

To further explore the influence of Kejinyan decoction on biological function of lung cancer, we carried out RNA sequence on tumor tissue isolated from Lewis lung carcinoma C57BL/6 mice treated with or without Kejinyan decoction and 13,343 common genes were identified between the two groups through a Venn diagram (Fig. 2a). To identify the genes with significant change in expression level caused by Kejinyan decoction treatment, differentially expressed transcripts (DETs) between the Mock group and the high dose of Kejinyan decoction group were identified, and 502 DETs were found between the two groups with at least |log2(FoldChange)|> 1, q value < 0.05, including 186 common up-regulated genes and 316 common down-regulated genes (Fig. 2b). Gene ontology (GO) functional classification analyses were performed to classify the functions of the DETs during Kejinyan decoction treatment. Based on sequence homology, all DETs could be categorized into 30 functional groups. In the biological process (BP) category, the more significantly regulated processes were the “immune system process” and the “immune response process” (*p* < 0.05 respectively). In the cellular component (CC) category, the greatest numbers of genes were found in the “extracellular region”. In the molecular function (MF) category, the “G-protein coupled receptor binding”, the “chemokine activity” and the “chemokine receptor binding” were also regulated significantly (*p* < 0.05 respectively) (Fig. 2c). Next, we conducted a KEGG-pathway-based analysis to further understand the biological functions of these DETs. According to the statistics of pathway enrichment, we screened out top 20 pathways, in which the “protein digestion and absorption”, “phagosome”, “nitrogen metabolism”, “focal adhesion”, “ECM-receptor interaction”, “cytokine–cytokine receptor interaction”, “calcium signaling pathway”, “antigen processing and presentation”, “arginine biosynthesis”, “arginine and proline metabolism” pathway etc. were included.

To determine whether genes involved in these pathways were enriched, the KEGG pathway database was searched using the DETs to reveal the top 20 significantly enriched pathways, and we found that the “cell adhesion”, “inflammatory mediator regulation”, “cytokine–cytokine receptor interaction”, “platelet activation”, “natural killer cell mediated cytotoxicity” and “energy associated pathway” were the key regulated pathway regulated by Kejinyan decoction. Besides, the “Metabolic pathways” were the more important pathway regulated by Kejinyan decoction, in which 18 pathways were down regulated and 10 pathways were upregulated.

### Effects of Kejinyan decoction on immunocyte subsets in the spleen of mice

**Fig. 3:**
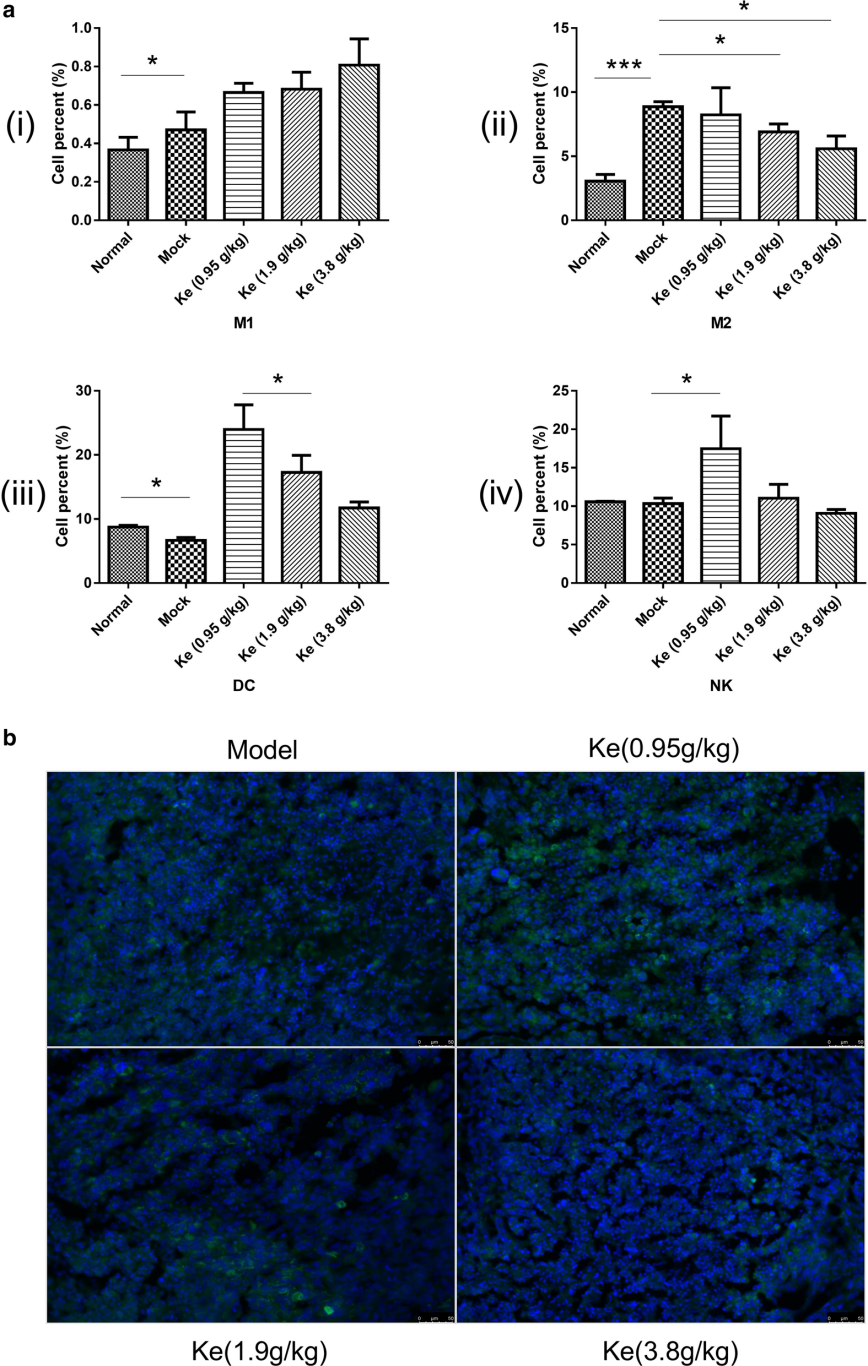
Effects of Kejinyan decoction on the immunocytes of the mice. **a** Quantification of NK, DC cells, and M1, M2 macrophages in the spleen of different mice groups. **b** Immunofluorescence detection of CD206 (200×). Data are presented as mean ± SEM. Unpaired *t*-test, *p < 0.05, ***p < 0.001

Then FCM was used to analyze leukocytes isolated from the spleens of the mice in different groups. For the macrophages, the number of M1 macrophages in the mock group increased significantly compared with that in the normal group (*p* < 0.05), and the levels of them showed an upward trend after treated with Kejinyan decoction (Fig. 3a(i)). Further detection of M2 cells showed that treatment of Kejinyan decoction significantly inhibited the level of M2 macrophage in a dose dependent manner (Fig. 3a(ii)). Meanwhile, we found that compared with the mock group the levels of DC and NK cells both increased significantly under the treatment of 0.95 g/kg Kejinyan decoction (*p* < 0.05) (Fig. 3a(iii) and (iv)) (FCM gating strategy as Additional file 1: Fig. S1).

TAMs and their precursors account for the largest fraction of the myeloid infiltrate in the majority of human solid malignancies. Especially, M2 type macrophages facilitate cancer progression by secreting cytokines to promote angiogenesis, immune-suppression, and metastasis [10]. Furthermore, we detected CD206 (the marker of M2 type macrophage) in the tumoral tissue sections by immunofluorescence assay respectively to further judge the effect of Kejinyan decoction on macrophage polarization. As shown in Fig. 3b, the levels of CD206 decreased significantly under the treatment of Kejinyan decoction.

### Effect of Kejinyan decoction on inflammatory level in Lewis lung cancer mice

Polarization of macrophage to either classically activated M1 macrophages or alternatively activated M2 phenotype largely depends on the cytokine milieu they exposed to. Usually, macrophages exposed to cytokines IL-12, TNF-α or IFN-γ acquire a pro-inflammatory (M1) state, while exposed to IL4, IL5, IL10, IL13, or TGF-β1 acquire an anti-inflammatory (M2) state [15].

**Fig. 4:**
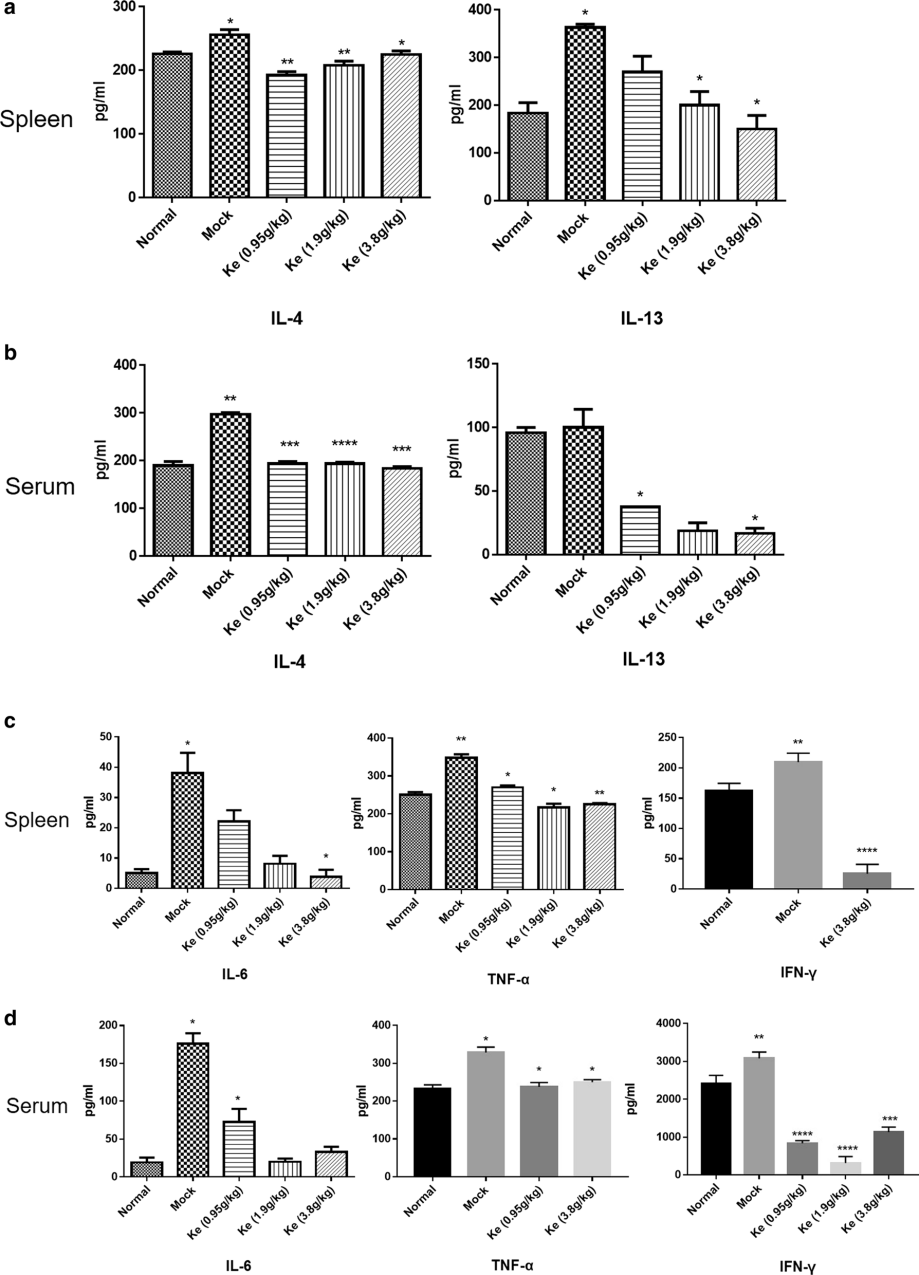
Effects of Kejinyan decoction on inflammatory cytokines in the Lewis lung cancer xenograft C57BALB/c mice. **a**, **b** The levels of IL-6, TNF-α and IFN-γ in mice spleen or serum were determined using ELISA assay. **c**, **d** The levels of IL-4 and IL-13 in mice spleen or serum were determined using ELISA assay. Values represented the mean ± SD (n = 6 per group), unpaired t test, *p < 0.05, **p < 0.01, and ***p < 0.001, ****p < 0.0001

We then detected the level of cytokines in the Lewis lung cancer mice treated with or without Kejinyan decoction to find out the effect of Kejinyan decoction on it. The results showed that in the spleen, the level of IL-4 and IL-13 increased significantly in the mock group compared with the normal group (*p* < 0.05), while treatment with 3.8 g/kg of Kejinyan decoction significantly inhibited the level of them (*p* < 0.05) (Fig. 4a). In the serum, the results of IL-13 were similar as that in the spleen samples,while the level of IL-4 was more significantly decreased by Kejinyan decoction than that in spleen samples (*p* < 0.001) (Fig. 4b).

Meanwhile, in the spleen, the level of the pro-inflammatory cytokines IL-6, TNF-α and IFN-γ in mock group mice all increased significantly compared with that in normal groups (*p* < 0.05, *p* < 0.01, *p* < 0.01, respectively), while Kejinyan decoction treatment downregulated the levels of them significantly, especially at the dose of 3.8 g/kg (*p* < 0.05, *p* < 0.01, *p* < 0.0001, respectively) (Fig. 4c). In the serum samples, the level of IFN-γ was dramatically inhibited after 0.95, 1.9, or 3.8 g/kg of Kejinyan decoction treatment (*p* < 0.0001, *p* < 0.0001, *p* < 0.001, respectively) by different concentrations of Kejinyan decoction (*p* < 0.001) (Fig. 4d).

### Detection of glucose metabolism in immune microenvironment

A large of evidence has proved that the induction of the hypoxia-responsive glucose transporter GLUT1 strongly correlates with oxygen shortage [16]. Recently, Jeong demonstrated clinically and preclinically that TAMs were a novel contributor to tumor hypoxia and aerobic glycolysis by competing oxygen and glucose with cancer cells [17].

Next, we observed F4/80 (the marker of total macrophage) and CD86 (the marker of M1) by confocal laser scanning microscope, meanwhile, the level of GLUT1 and FBP1 were also detected to find out the hypoxia level and glycolysis level in the tumor.

**Fig. 5:**
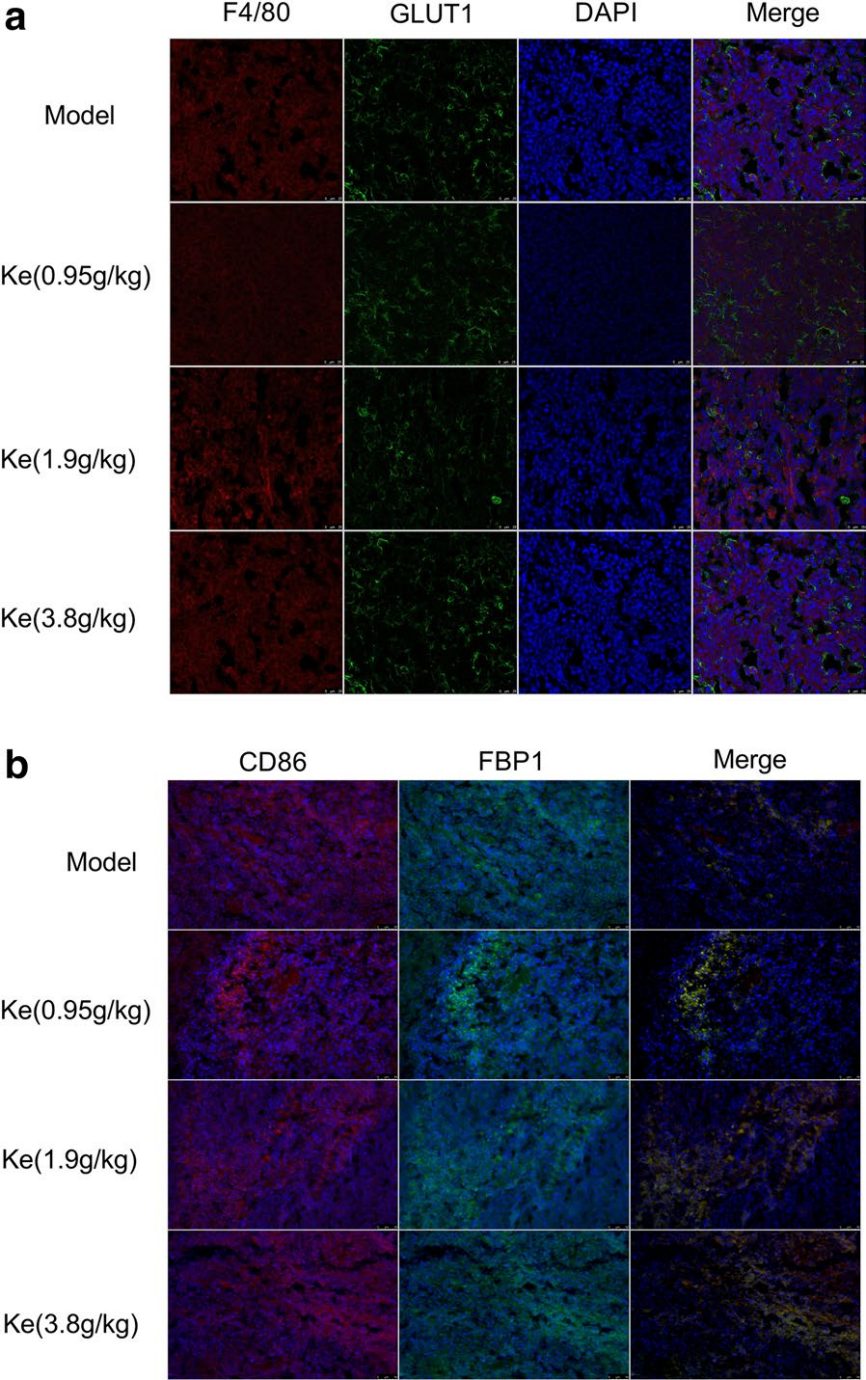
Effects of Kejinyan decoction on the level of macrophages and glucose metabolism in the Lewis lung cancer xenograft C57BL/6 mice. **a** Representative images of F4/80 and GLUT1 staining (TAMs) in LLC tumors (400×). **b** Representative images of CD86 and FBP1 staining (TAMs) in LLC tumors (200×)

We found that the level of F4/80 and GLUT1 both decreased significantly in the tumors treated by Kejinyan decoction (Fig. 5a), which mean that Kejinyan decoction decreased levels of tumor-infiltrating macrophages, meanwhile decreased glucose consumption in tumors. Otherwise, the level of CD86 and FBP1 both increased (Fig. 5b), which mean that Kejinyan decoction treatment increased the level of M1 while decreased the level of glycolysis in M1 cells.

Together, Kejinyan decoction reduced the level of tumor infiltrating macrophages, and promoted the phenotype of macrophages to M1 type. In addition, it reduced the glucose metabolism level of tumor microenvironment, which may be the reason of inhibiting migration.

### Influence of Kejinyan decoction on the metastatic ability of Lewis lung cancer cells in vivo

**Fig. 6:**
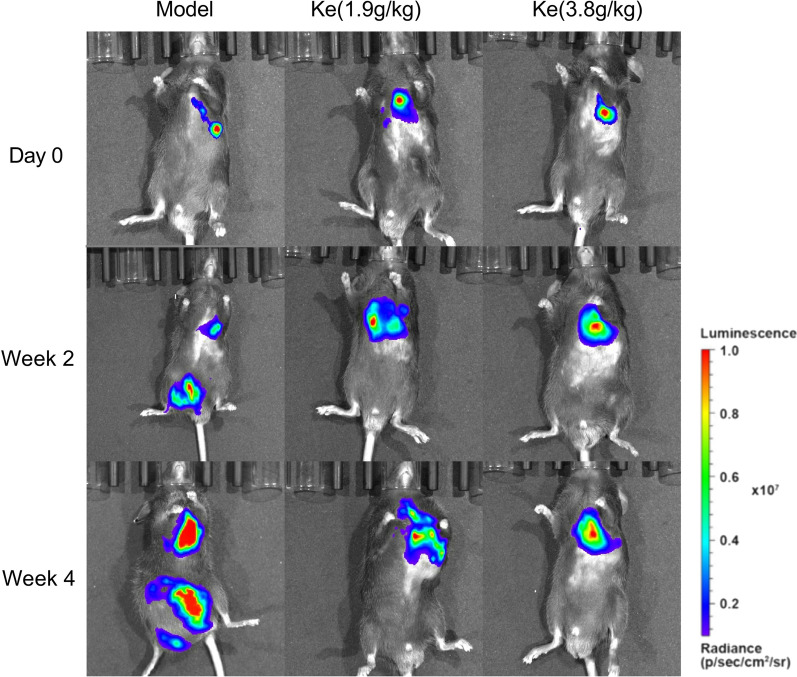
Influence of Kejinyan decoction on the metastatic ability of Lewis lung cancer cells in vivo. Bioluminescent imaging of Kejinyan decoction influenced groups with left lung parenchyma injection of luciferase-marked Lewis lung cancer cells

To assess the influence of Kejinyan decoction on the metastatic ability of Lewis lung cancer cells in vivo, we established a Lewis lung cancer orthotopic xenograft tumor model. In bioluminescent images, dissemination and metastasis in the luciferase tracking Lewis lung cancer cells were both suppressed by Kejinyan decoction, especially at the concentration of 3.8 g/kg (Fig. 6).

## Discussion

Tumor hypoxia and glycolysis have long been recognized as major resistance factors contributing to failures of chemo-and radiotherapy, while TAMs are usually recruited toward low-oxygen-tension regions [18, 19]. Jeong et al*.* recently found a strong correlation between CD68 TAM immunostaining and PET 18 fluoro-deoxyglucose (FDG) uptake in 98 matched tumors of patients with non-small cell lung cancer (NSCLC). They also demonstrated that TAM secreted TNF-α to promote tumor cell glycolysis, whereas increased AMP-activated protein kinase and peroxisome proliferator-activated receptor gamma coactivator 1-alpha in TAM facilitated tumor hypoxia [17]. Moreover, reprogrammed metabolism has emerged as a hallmark of malignancies and plays an important role in cancer development [14], and the impact of TAMs on tumor progression very likely depends on their specific reprogramming within the tumor, a process influenced by TME [20–23].

Our previous investigation proved that Kejinyan decoction had significant anti-lung cancer effect. More importantly, it significantly improved the quality of life of the mice compared with the cisplatin treatment [12]. In this study, we carried out the experiments on C57BL/6 Lewis lung cancer mice, and found that the overall survival time of the mice prolonged significantly after intragastric administration Kejinyan decoction for several weeks, especially under the concentration of 3.8 g/kg (Fig. 1a and Additional file 2: Fig. S2).

Firstly, we carried out RNA-sequencing on the tumor tissue to reveal the regulation effects on the biology of tumor microenvironment system, and analysis of the results showed that Kejinyan decoction-administration significantly regulated immune response and chemokine activity (Fig. 2). Most importantly, the KEGG pathway database showed that “metabolic pathways” were the most important pathway regulated by Kejinyan decoction, in which 28 pathways were regulated, mainly including carbohydrate metabolism pathways.

Further detection of immune cells in the spleen of the mice by flow cytometry found that treatment of Kejinyan decoction significantly inhibited the level of M2 macrophage in a dose dependent manner (Fig. 3a), and detection of CD206 (the marker of M2 phages) by immunofluorescence assay also verified the results (Fig. 3b). Meanwhile, the level of F4/80 was observed to be decreased by Kejinyan decoction treatment, which meant that Kejinyan decoction treatment decreased the level of tumor-infiltrating macrophages (Fig. 5a).

Inflammation is the important characteristic of cancer [14, 20], and TAMs are the key cells linking tumor and inflammation, and also the main effector cells of cancer related inflammation [20, 24]. On the contrary, the production of different types of inflammatory factors in TME also affects the polarization of TAMs [4], after alternatively activate to M2 phenotype, TAMs play important role in promoting metastasis, invasion and immune escape of tumor cells [25, 26]. To find out whether Kejinyan decoction change the inflammatory microenvironment of the mice, we detected a series of inflammatory factors such as TNF-α, IFN-γ, IL-6, IL-4, and IL-13 in the spleen and serum, and found that Kejinyan decoction inhibited all of them significantly, especially on IFN-γ (Fig. 4). As an inflammatory factor in tumor microenvironment, IFN-γ is one of the main factors inducing overexpression of PD-L1 in tumor cells and resulted in immune escape [27, 28].The underlying mechanism is that IFN-γ induces PD-L1 expression by activating downstream JAK-STAT signaling pathway [29, 30]. So, whether Kejinyan decoction can inhibit immune escape of tumor cells by inhibiting IFN-γ is worth further exploring.

Moreover, metabolic pathways in immune cells also altered their function [3]. In the metabolic pathways involved in immune-metabolism, increased glycolysis was considered a hallmark metabolic change in most immune cells undergoing rapid activation, for macrophages this includes phagocytosis and inflammatory cytokine production [31, 32]. Further detection of hypoxia-related index GLUT1 showed that the expression of GLUT1 decreased significantly after treatment of Kejinyan decoction (3.8 g/kg), which indicated that Kejinyan decoction may decrease hypoxia in tumors (Fig. 5a). Meanwhile, detection results of M1 macrophage surface marker CD86 and glycolysis related index FBP1 showed that Kejinyan decoction significantly increased the polarization level of M1 macrophages and decreased the glycolysis level (Fig. 5b). Furthermore, it inhibited the migration of Lewis lung cancer cells in vivo (Fig. 6)*.*

Taken together, through decreasing the level of inflammatory factors and reprogramming glucose metabolism, Kejinyan decoction improved tumor microenvironment, which resulted in the change of the biological characteristics of macrophages and tumor cells. The correlated molecular mechanisms still remains to be further studied.

## Conclusion

Despite our incomplete understanding of mechanisms involved in the anti-lung cancer effects of Kejinyan decoction, the results of this study clearly demonstrate that Kejinyan decoction inhibited Lewis lung cancer cell metastasis through inhibiting the level of inflammation, affecting macrophage polarization and energy reprogramming. However, how Kejinyan decoction affects the migration of cancer cells in vivo by altering the polarization levels of M1 and M2 and their respective metabolic reprogramming remains to be further studied.

## Supplementary information

**Additional file 1: Fig. S1.** Effects of Kejinyan decoction on the immunocytes of the mice, FCM gating strategy.

**Additional file 2: Fig. S2.** Survival time of the mice treated with different concentrations of Kejinyan decoction.

## Data Availability

The datasets used and/or analyzed during the current study are available from the corresponding author on reasonable request.

## Abbreviations

LLC: Lewis lung cancer
TME: Tumor microenvironment
TAMs: Tumor-associated macrophages
RNA-Seq: RNA-sequence
ELISA: Enzyme-linked immunosorbent assay
GO: Gene ontology
TCM: Traditional Chinese Medicine
SPF: Specific pathogen-free
IFN-γ: Interferon-γ
TGF- β: Transforming growth factor-β
TNF-α: Tumor Necrosis Factor alpha
IL: Interleukin
FCM: Flow cytometry
DETs: Differentially expressed transcripts
GLUT1: Glucose transporter 1
