# Supplementary figures and images for: Study on metastasis inhibition of Kejinyan decoction on lung cancer by affecting tumor microenvironment
Source: Cancer Cell Int. 2020 Sep 14;20:451. doi: 10.1186/s12935-020-01540-0 (PMC7490898; doi:10.1186/s12935-020-01540-0)

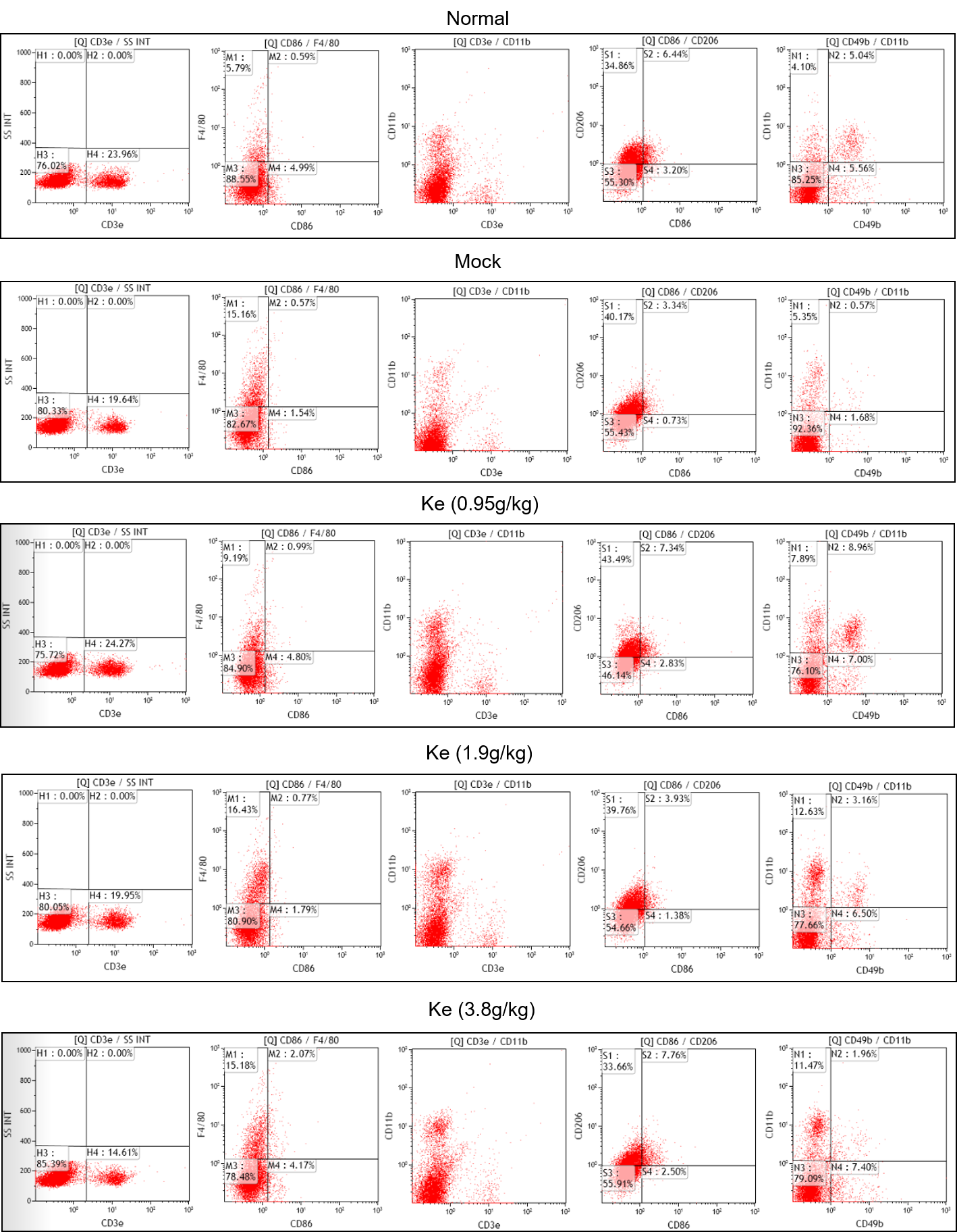

Supplement: Supplementary file 1 — Additional file 1: Fig. S1. Effects of Kejinyan decoction on the immunocytes of the mice, FCM gating strategy. [file 12935_2020_1540_MOESM1_ESM.tif]
